# Kruppel-like factor 8 regulates triple negative breast cancer stem cell-like activity

**DOI:** 10.3389/fonc.2023.1141834

**Published:** 2023-04-19

**Authors:** Giang Le Minh, Emily M. Esquea, Tejsi T. Dhameliya, Jessica Merzy, Mi-Hye Lee, Lauren E. Ball, Mauricio J. Reginato

**Affiliations:** ^1^ Department of Biochemistry and Molecular Biology, Drexel University College of Medicine, Philadelphia, PA, United States; ^2^ Department of Cell and Molecular Pharmacology and Experimental Therapeutics, Medical University of South Carolina, Charleston, SC, United States; ^3^ Translational and Cellular Oncology Program, Sidney Kimmel Cancer Center of Thomas Jefferson University, Philadelphia, PA, United States

**Keywords:** KLF8, cancer stem cell, self-renewal, breast cancer, OGT, O-GlcNAc

## Abstract

**Introduction:**

Breast tumor development is regulated by a sub-population of breast cancer cells, termed cancer stem-like cells (CSC), which are capable of self-renewing and differentiating, and are involved in promoting breast cancer invasion, metastasis, drug resistance and relapse. CSCs are highly adaptable, capable of reprogramming their own metabolism and signaling activity in response to stimuli within the tumor microenvironment. Recently, the nutrient sensor O-GlcNAc transferase (OGT) and O-GlcNAcylation was shown to be enriched in CSC populations, where it promotes the stemness and tumorigenesis of breast cancer cells in vitro and in vivo. This enrichment was associated with upregulation of the transcription factor Kruppel-like-factor 8 (KLF8) suggesting a potential role of KLF8 in regulating CSCs properties.

**Methods:**

Triple-negative breast cancer cells were genetically modified to generate KLF8 overexpressing or KLF8 knock-down cells. Cancer cells, control or with altered KLF8 expression were analyzed to assess mammosphere formation efficiency, CSCs frequency and expression of CSCs factors. Tumor growth in vivo of control or KLF8 knock-down cells was assessed by fat-pad injection of these cell in immunocompromised mice.

**Results:**

Here, we show that KLF8 is required and sufficient for regulating CSC phenotypes and regulating transcription factors SOX2, NANOG, OCT4 and c-MYC. KLF8 levels are associated with chemoresistance in triple negative breast cancer patients and overexpression in breast cancer cells increased paclitaxel resistance. KLF8 and OGT co-regulate each other to form a feed-forward loop to promote CSCs phenotype and mammosphere formation of breast cancer cells.

**Discussion:**

These results suggest a critical role of KLF8 and OGT in promoting CSCs and cancer progression, that may serve as potential targets for developing strategy to target CSCs specifically.

## Introduction

Breast cancer is a major burden for women world-wide, being the most common cancer and the leading cause of cancer-related death in women (1). Breast cancer is classified into several sub-types based on expression of hormone and growth factor receptors (2). Among them, the triple-negative breast cancer (TNBC) is the most aggressive breast cancer form with no current targeted treatment, poor survival rate, high risk of metastatic spread and short recurrence-free interval (3, 4). The growth of breast tumor cells, metastasis and recurrence are highly influenced by a population of breast cancer cells, the cancer stem-like cells (CSCs) (5). Unlike other tumor cells, CSCs are capable of self-renewing and differentiating (6) to promote and maintain tumor growth, as well as the intra-tumor heterogeneity (7). Importantly, CSCs are highly resistant to chemotherapy, resulting in chemoresistance and tumor relapse (8). Therefore, targeting CSCs in combination with conventional chemotherapy is of interest in finding new approach to treat breast cancer.

The enzyme O-GlcNAc transferase (OGT) (9), which is responsible for adding GlcNAc moiety from UDP-GlcNAc on serine/threonine residues of target nuclear/cytoplasmic proteins (10) is a key metabolic sensor, linking alteration in nutrient status and signaling pathways in cancer. This protein modification is termed O-GlcNAcylation (11). O-GlcNAcylation, along with OGT are found to be highly elevated in a variety of cancers including breast cancer (12). OGT and O-GlcNAcylation can regulate many hallmarks of tumor progression (9). Recent studies have linked OGT/O-GlcNAc signaling to CSCs properties in various cancers (13–18). Specifically, elevated OGT/O-GlcNAcylation is sufficient to drive CSCs properties of breast cancer cells (18), as well as lung and colon cancer cells (13, 14, 18).

Kruppel-like factor 8 (KLF8) is a transcription factor belonging to the Kruppel-like transcription factor family (19). KLF8 has been shown to directly bind and suppress expression of epithelial cell marker E-Cadherin (20), thus promoting the epithelial-mesenchymal transition (21, 22). Importantly, KLF8 expression is elevated in various cancers, and is associated with increased invasiveness and metastasis (22–24). However, the role of KLF8 in CSCs and tumor initiation remains unknown. Recently, we observed increased levels of KLF8 in CSCs-enriched mammospheres, and increased OGT/O-GlcNAcylation upregulated KLF8 level in breast cancer cells, both *in vitro* and *in vivo* (18), suggesting a potential role of KLF8 in regulating CSCs properties in breast cancer cells.

Here, we show that overexpression of KLF8 in TNBC cells increased mammosphere formation and CSC population, while genetic targeting of KLF8 expression reduced mammosphere formation and CSC population. KLF8 regulated mRNA and protein levels of CSCs markers, including OCT4, SOX2, MYC and NANOG in breast cancer cells. Moreover, increased KLF8 expression increased resistance of TNBC cells to paclitaxel-induced apoptosis. Interestingly, KLF8 and OGT coregulate each other and may form a feed-forward loop in breast cancer cells. Lastly, reducing KLF8 expression also blocked TNBC cell growth *in vivo*. Together, our data suggests a potential role of KLF8 in a feed-forward loop with OGT to regulate CSCs properties, as well as resistance to chemotherapeutic agents. KLF8 and OGT may serve as promising targets to overcome challenges in treating breast cancer.

## Materials and methods

### Cell lines

Human TNBC cell line MDA-MB-231 was purchased from the ATCC (American Type Culture Collection, Manassas, VA, USA), and was cultured in humidified condition with 5% CO_2_ in complete medium Dulbecco’s Modification Eagle’s Medium with 4.5g/L glucose (DMEM, Genesee), supplemented with 10% fetal bovine serum (FBS, Gemini), 1% L-Glutamine (Gibco/Life-Technologies), 1% penicillin/streptomycin (Invitrogen). Human TNBC patient-derived xenograft cells HCI-10 were received as a kind gift from Dr. Seagroves (University of Tennessee), and were cultured in a humidified atmosphere with 5% CO2 in DMEM/F12 supplemented with 2% endotoxin-low FBS (Gibco), 1x insulin-transferrin-selenium (ITS) (Corning), 1x penicillin-streptomycin (Gibco), human epidermal growth factor (5 ng/mL), hydrocortisone (0.3 μg/ml), cholera toxin (0.5 ng/mL) 3,3’,5-triiodo-L-thyronine (5 nM), isoproterenol hydrochloride (5 μM), ethanolamine (50 nM), O-phosphorylethanolamine (50 nM) and HEPES (10 mM). TNBC cell line SUM159 was received as a kind gift from Dr. Seagroves (University of Tennessee) and was cultured in the same condition as described above, in DMEM/F12 medium supplemented with 10% fetal bovine serum (FBS, Gemini), 1% L-Glutamine (Gibco/Life-Technologies), 1% penicillin/streptomycin (Invitrogen), 0.1% Insulin and 0.05% Hydrocortisone. TNBC cells stably overexpressing KLF8 (GeneCopoiea, EX-T1091-Lv181-KLF8) or OGT (GeneCopoeia, EX-Z3428-M13-10-OGT), were generated through lentiviral transduction. All cells were regularly tested for mycoplasma contamination.

### RNA interference

KLF8 knockdown was performed using two different lentiviral constructs, purchased from Sigma-Aldrich (TRCN0000015881, TRCN0000015878). The sequences of shRNA encoded in these constructs are: CCGGCCTCAGTCAGTCTGCCAAATACTCGAGTATTTGGCA-GACTGACTGAGGTTTTT (KLF8 shRNA-1) and CCGGCGCTGCTTTATTCTTTCCAA-TCTCGAGATTGGAAAGAATAAAGCAGCGTTTTT (KLF8 shRNA-2). OGT knockdown was generated using two different lentiviral constructs from Sigma-Aldrich. The sequence of shRNA encoded in these constructs are: GCCCTAAGTTTGAGTCCAAATCTCGAGAT-TTGGACTCAAACTTAGGGC (OGT shRNA-1) and GCTGAGCAGTATTCCGAG-AAACTCGAGTTTCTCGGAATACTGCTCAGC (OGT shRNA-2). Control shRNA was acquired through Addgene (plasmid 1864), from Dr. Sabatini (Whitehead Institute for Biomedical Research, MIT) and the sequence used was: CCTAAGGTTAAGTCGCC-CTCGCTCGAGCGAGGGCGACTTAACCTTAGG. pLKO-Puro vectors carrying control (scramble sequence) shRNA, and KLF8 shRNA sequences (KLF8-1 or KLF8-2) shRNA were packaged into VSVG-pseudotyped lentiviruses, through co-transfection of HEK-293T packaging cells with 10 µg of vector DNA and appropriate packaging vectors. Stable cell lines for shRNA knockdowns were generated as previously described (25).

### Mammosphere formation assay

In mammosphere formation assay, a predetermined amount of cells (200 cells for MDA-MB-231 and HCI-10, or 800 cells for SUM159) were cultured in 96-well plate, precoated with 1.2% polyHEMA in absolute ethanol to prevent cells adherence to plate surface. Cells were cultured in DMEM/F12 (Gibco), 20ng/ml EGF (Sigma), 20ng/ml bFGF (Invitrogen: PHG0024), B27 50x (Invitrogen; 17504-044), and 1mg/ml Pen/Strep (Gibco) for 5-7 days. Mammosphere greater than 50μm were counted and mammosphere formation efficiency (MFE) was calculated using the following formula: (number of mammospheres/number of cells plated) * 100%. For secondary mammospheres, mammospheres cultured in conditions described above were collected and resuspended to make single cell suspensions. A predetermined number of cells from retrieved suspensions (200 cells for MDA-MB-231 and HCI-10, or 800 cells for SUM159) were cultured in poly-HEMA precoated 96-well plates in mammosphere culture conditions as described above. After 5-7 days, mammosphere greater than 50µm were counted and mammosphere formation efficiency for secondary mammosphere were determined using the formula described above.

### Flow cytometry

In NANOG-GFP reporting assay, cells were stably infected with lentivirus containing construct coding for eGFP under control of NANOG promoter (Addgene). Cells were then collected by treating with 0.25% Trypsin with EDTA, followed by PBS washing. Collected cells were counted and resuspended in PBS to cell concentration of 4×10^5^/mL. 500μL of cells suspension were then analyzed by flow cytometry system Guava-easyCyte (Millipore). Control cells without NANOG-GFP infection were used as negative control to determine the basal fluorescent signal. Cells were gated by Forward-scatter and Side-scatter, followed by green fluorescence emission. All data were collected and analyzed using Guava EasyCyte Plus System and CytoSoft (version 5.3) software (Millipore). The ALDEFLOUR assay kit (ALDH assay) was obtained from STEMCELL Technologies. ALDH assay was performed following the provided instructions. In brief, cells were trypsinized and counted. 2×10^5^ cells were washed twice with PBS and resuspended in 1mL of provided ALDEFLOUR assay buffer. 5μL of activated BAAA-DA was added to cell suspension. Immediately after adding BAAA-DA, 500μL of the mixture were quickly transferred to another tube, containing 5μL of DEAB reagent, an inhibitor of ALDH. All samples were incubated for 30 minutes at 37°C. After incubation, cells were pelleted and resuspended in 500μL of provided assay buffer. Green fluorescence signal was detected and analyzed using flow cytometry system Guava-easyCyte (Millipore) as per manufacturer’s instructions. In brief, the control sample with DEAB inhibiting ALDH activity was used to determine the basal fluorescence emission. The higher %ALDH+ signifies the percentage of cells that have high ALDH activity.

### Western blotting

Cells were collected and washed three-times with ice-cold PBS to remove residue media. Then cell pellets were resuspended in cold RIPA lysis buffer (150mM NaCl, 1% NP40, 0.5% DOC, 50mM Tris-HCl at pH 8, 0.1% SDS, 10% glycerol, 5mM EDTA, 20mM NaF and 1mM Na3VO4), supplemented with protease inhibitors. Cell debris was removed by centrifugation at 15000 rpm for 20 minutes at 4°C using benchtop centrifuge. Protein concentration was determined flowing Bradford assay. 50 µg of protein from each sample was separated by SDS-PAGE and transferred to PVDF membrane. Target proteins were detected using indicated specific antibodies: Anti-OGT (Cell-Signaling, cat #20438), Anti-O-GlcNAc (Sigma, cat #MABS1254), Anti-c-MYC (Novus, cat #NB600-335), Anti-Actin (Santa Cruz, cat #sc-47778), Anti-NANOG (Cell-Signaling, cat #3580), Anti-SOX2 (Cell-Signaling, cat #3579), Anti-OCT4 (Cell-Signaling, cat #2750), Anti-KLF8 (Sigma, cat #AV32859).

### Quantitative RT-PCR

Total RNA from TNBC cells was extracted using Trizol reagent (Invitrogen) following manufacturer’s instructions. In brief, cells in monolayer were lysed and collected in Trizol reagent. Total RNA was fractionated by chloroform extraction. RNA samples were then precipitated using isopropanol and washed extensively with 70% ethanol. After ethanol wash, RNA was air-dried and re-dissolve in nuclease-free water. mRNA level of target genes was determined using Brilliant II qRT-PCR Master Mix Kit (Agilent) following manufacturer’s instruction using qRT-PCR Applied Biosystems 7500 (Applied Biosystem). Following Taqman probes (Applied biosystem) were used to determine target gene expression: KLF8 (Hs06664122_s1), PPIA (Hs04194521_s1), OCT4 (Hs04260367), SOX2 (Hs04234836_s1), c-MYC (Hs00905030_m1), Nanog (Hs02387400_g1) and OGT (Hs00269228_m1).

### Apoptosis assay

TNBC cells in monolayer were treated with increasing dose of paclitaxel for 48h. DMSO was used as control. After 48h, cells were collected, counted and washed with PBS. Cell apoptosis was assessed using Annexin V-FITC Apoptosis detection kit (Biolegend) following manufacturer’s instructions. In brief, 10^6^ cells were resuspended in 1mL of 1X provided binding buffer. 100μL cells suspension were then incubated with 5μL Annexin-V and 5μL Propidium Iodide for 15 minutes in the dark, following by flow cytometry analysis. Unstained and single stained cells were used as controls for determining basal fluorescence signal.

### Clonogenic survival assay

TNBC cells growing in monolayer were treated with increasing dose of paclitaxel as described above. After 48h, cells were collected and counted. 1000 cells from each condition were plated into a 6-well plate with fresh media and resuspended to avoid cell clusters. Cells were allowed to grow for 10-14 days before staining with crystal violet (Sigma). Colonies were counted and normalized against control without paclitaxel treatment.

### Animal studies

SUM159 cells stably expressing luciferase were generated by transducing with pWZL-Hygro-luciferase (kindly provided by Dr. Maureen Murphy, Wistar Institute). KLF8 knockdown were generated using lentivirus containing KLF8 shRNA, non-target shRNA was used as control. Cells were injected to fat pad of nude mice as described before (26). In brief, cells were collected and counted. 1.5×10^6^ cells in 200μL PBS with 20% Matrigel (Corning) were injected to fat pad of 6-8 weeks old female nude mice. Mice were imaged weekly for bioluminescence *via* intraperitoneal injection of D-Luciferin 10uL/g of body weight (30mg/ml in DPBS: Perkin Elmer) on the IVIS 200. At 8 weeks post-injection, mice were euthanized by CO2 and tumors were taken out and weighted, as well as measured using digital calipers. Tumor volume was calculated using the formula (LxW^2^)/2. Mouse experiments were performed with the approval of Institutional Animal Care and Use Committee.

### Database analysis

Kaplan-Meier plots were generated using available online database Kaplan-Meier plotter (https://kmplot.com/analysis) (27). Relevance KLF8 mRNA expression in total breast cancer (auto select best cutoff, overall survival) and in basal-type breast cancer (auto select best cutoff, overall survival, StGallen subtype: basal) were plotted against the overall survival of breast cancer patients.

Relative gene expression of KLF8 and relevant ROC plot in breast cancer patients with different relapse-free five years survival toward taxane (relapse-free survival at 5 years, taxane) was plotted using available database ROC plotter (https://www.rocplot.org/site/treatment) (28).

### Statistical analysis

Results shown are from at least three independent experiments presented as mean ± SEM. Represents statistical significance with p-value <0.05, p<0.01 and p<0.001 as indicated.

## Results

### KLF8 levels regulate breast cancer cells stem cell properties

**Figure 1:**
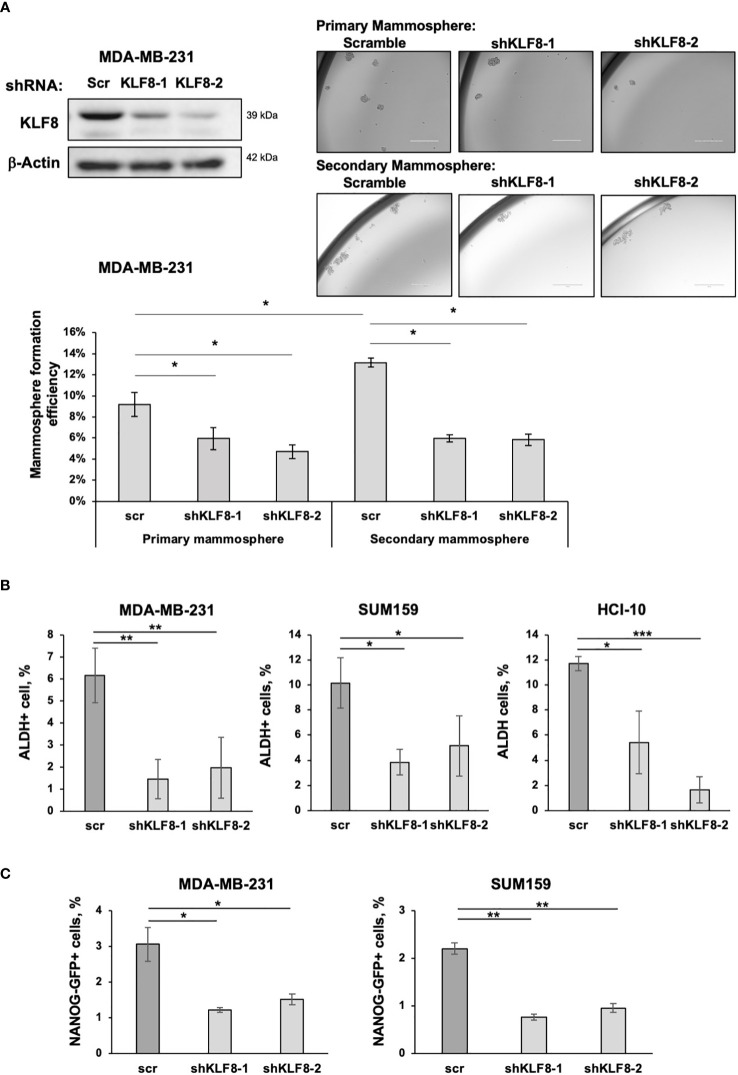
KLF8 inhibition reduced mammosphere formation and CSCs population in TNBC cells. **(A)** Lysates from MDA-MB-231 cells stably expressing control or KLF8 shRNA was collected for immunoblot analysis using antibodies against indicated proteins (top-left). MDA-MB-231 cells transduced with control or KLF8 shRNA were grown in mammosphere formation assay for 5-7 days. Representative images of mammosphere were taken (top-right) (scale bar 400 μm), and mammosphere larger than 50µm were counted and primary mammosphere formation efficiency was quantified by fractionating counted mammosphere number from total cells plated at the beginning of the assay. Primary mammosphere were then collected and culture again in mammosphere culture condition for 5-7 days to form secondary mammosphere (bottom). **(B)** Quantified graph of ALDH+ CSCs population detected by flow cytometry from MDA-MB-231, SUM159 and PDX cells HCI-10 stably expressing control or KLF8 shRNA. **(C)** Quantified graph showing NANOG-GFP+ CSCs population detected by flow-cytometry from MDA-MB-231 and SUM159 cells stably expressing control or KLF8 shRNA. Student *t* test reported as mean ± SEM, *p<0.05, **p<0.01, and ***p<0.001.

We previously identified a critical role of OGT and O-GlcNAc in promoting CSCs phenotype and tumor initiation, potentially *via* upregulating KLF8 expression (18). Since the role of KLF8 on breast cancer stem cells has not been examined, we tested the role of KLF8 in regulating stem cells properties in breast cancer cells. We utilized shRNA targeting KLF8 to reduce KLF8 expression in TNBC cells, MDA-MB-231, and assessed mammosphere formation efficiency. Mammosphere formation is highly influenced by the abundance of CSCs in the initial breast cancer cell population (29). Knockdown of KLF8 expression significantly impaired primary mammopshere formation in MDA-MB-231 cells (**Figure 1A**). We also tested secondary mammosphere formation by dissociating primary mammospheres generated in the presence of control or KLF8 RNAi into single cells and placed in secondary mammosphere assays. KLF8 RNAi expressing cells did not form secondary mammospheres as efficiently as cells from control mammospheres (**Figure 1A**), suggesting KLF8 may play a role in self-renewal. Similarly, the primary and secondary mammosphere formation efficiency in TNBC cells SUM159 (**Supplemental Figure 1A**) and patient-derived xenograft (PDX) cells HCI-10 (30) (**Supplemental Figure 1B**) with KLF8 knockdown was significantly reduced compared to control shRNA. To further evaluate the role of KLF8 in regulating CSCs population, we used two well established methods of detecting CSC population by measuring activity of aldehyde dehydrogenase (ALDH) (31), or by using NANOG-GFP reporter (32). Knockdown of KLF8 expression significantly reduced level of ALDH+ cells in MDA-MB-231, SUM159 and HCI-10 cells compared to control cells (**Figure 1B**). Consistent with this data, we also detected a significant decrease in NANOG-GFP+ cells in breast cancer cells MDA-MB-231 and SUM159 stably expressing KLF8 RNAi compared to control cells (**Figure 1C**). This data suggests that KLF8 is necessary in regulating CSCs phenotypes in TNBC cells.

**Figure 2:**
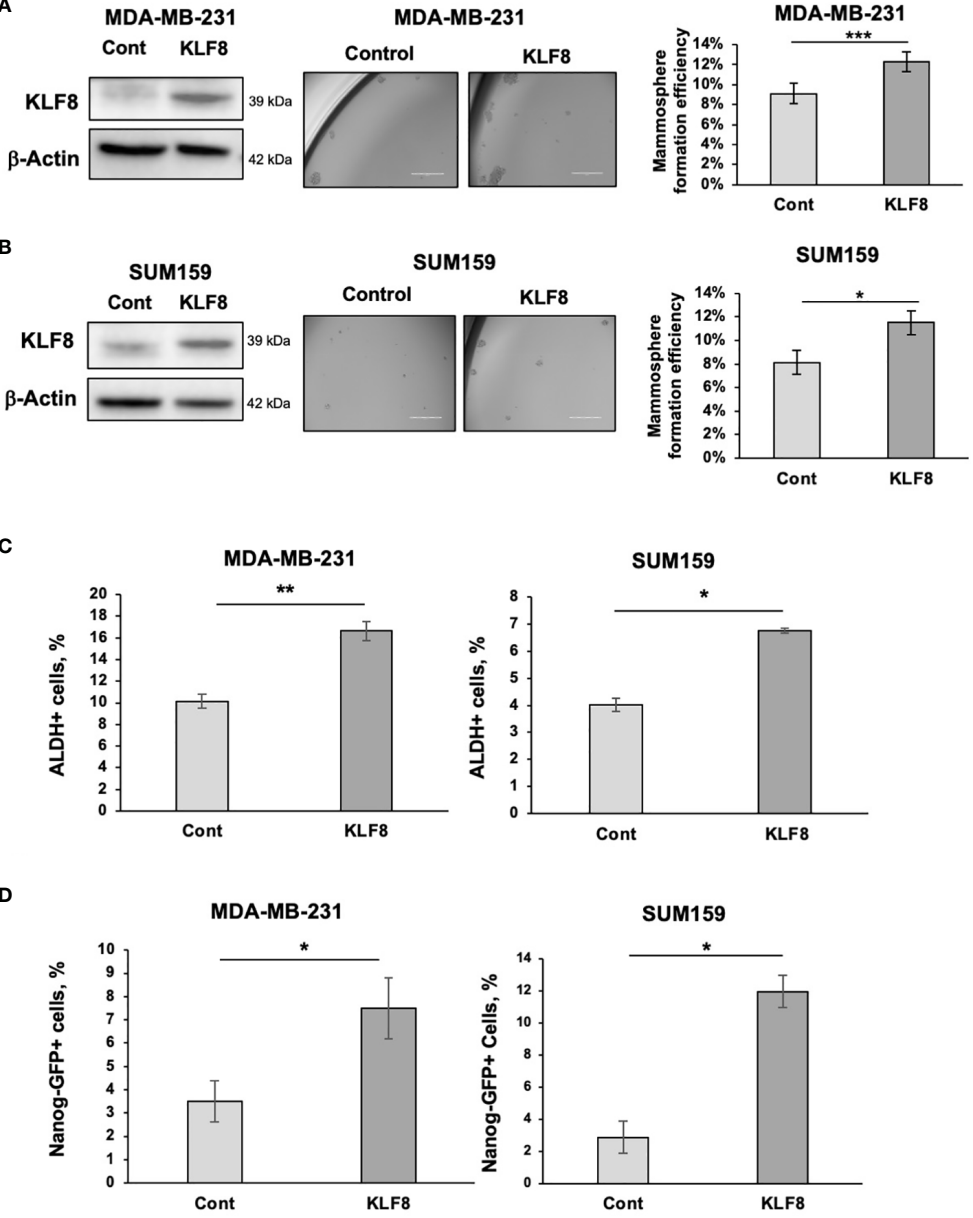
Elevated KLF8 expression increased mammosphere formation and CSCs population in TNBC cells. **(A)** Lysates from MDA-MB-231 cells stably overexpressing control or KLF8 were collected for immunoblot analysis using antibodies against indicated antibodies (left). MDA-MB-231 cells overexpressing control or KLF8 was grown in mammospheres formation assay for 5-7 days. Representative images of mammosphere were taken (middle) (scale bar 400 μm) and mammosphere larger than 50µm were counted and mammosphere formation efficiency was quantified and graphed (right). **(B)** Lysates from SUM159 cells overexpressing control or KLF8 were collected for immunoblot analysis using indicated antibodies (left). SUM159 cells overexpressing control or KLF8 was grown in mammospheres formation assay for 5-7 days. Representative images of mammosphere were taken (middle) (scale bar 400 μm) and mammosphere larger than 50µm were counted and mammosphere formation efficiency was quantified and graphed (right). **(C)** Quantified graph of ALDH+ CSCs population detected by flow cytometry from MDA-MB-231 and SUM159 cells overexpressing control or KLF8. **(D)** Quantified graph of NANOG-GFP+ CSCs population detected by flow cytometry from MDA-MB-231 and SUM159 cells overexpressing control or KLF8. Student *t* test reported as mean ± SEM, *p<0.05, **p<0.01, and ***p<0.001.

To test whether KLF8 expression is sufficient to increase stemness in breast cancer cells, we stably overexpressed KLF8 in TNBC cells MDA-MB-231 and SUM159 using lentiviral vectors. Overexpression of KLF8 significantly enhanced the mammosphere formation efficiency in both MDA-MB-231 (**Figure 2A**) and SUM159 cells (**Figure 2B**). Similarly, the population of ALDH+ (**Figure 2C**) and NANOG-GFP+ (**Figure 2D**) CSCs in KLF8 overexpressing cancer cells was significantly increased compared to control cells. Together, these data suggest that in triple negative breast cancer cells, KLF8 expression is necessary and sufficient to promote CSCs-phenotypes *in vitro*.

### KLF8 regulates cancer stem cell factors

**Figure 3:**
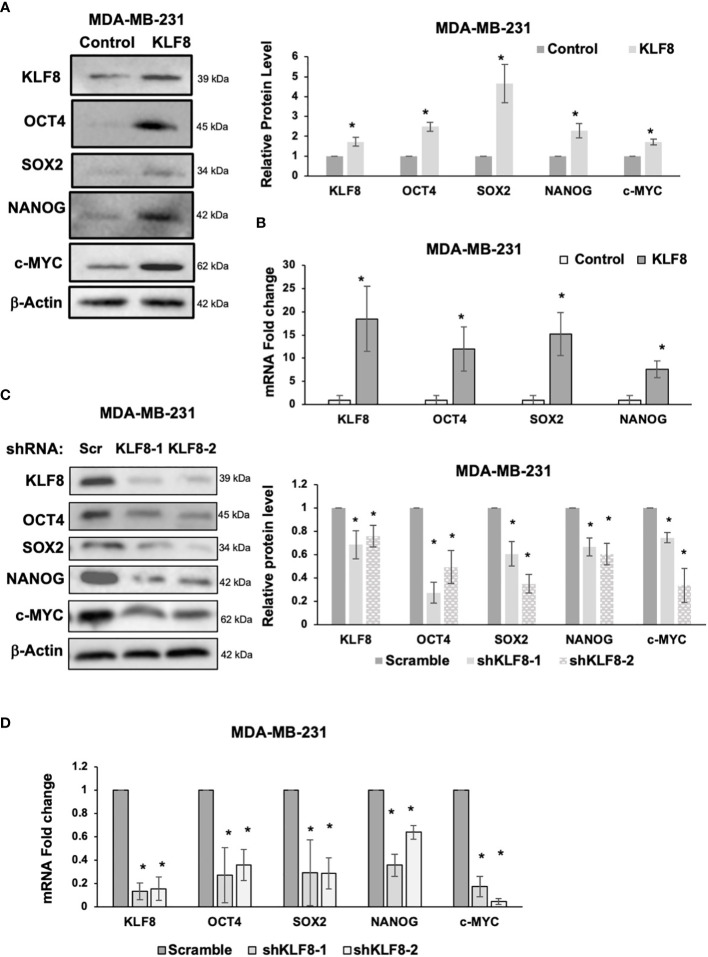
KLF8 regulates expression of stem cell markers in breast cancer cells. **(A)** Lysates from MDA-MB-231 cells stably overexpressing control or KLF8 were collected for immunoblot analysis of CSCs markers using indicated antibodies (left), and quantified graph of relative level of CSCs detected by immunoblot from MDA-MB-231 cells control or with KLF8 overexpression (right). **(B)** Quantified graph showing relative mRNA level of CSCs markers as detected by qRT-PCR using probes against genes in MDA-MB-231 cells stably overexpressing control or KLF8. **(C)** Lysates from MDA-MB-231 cells stably expressing control or KLF8 shRNA were collected for immunoblot analysis of CSCs markers using indicated antibodies (left), and quantified graph of relative level of CSCs levels (right). **(D)** Quantified graph showing relative mRNA level of CSCs markers as detected by qRT-PCR using probes against indicated targets in MDA-MB-231 cells stably expressing control or KLF8 shRNA. Student *t* test reported as mean ± SEM, *p<0.05.

Because CSCs population in breast cancer cells were enriched by KLF8 overexpression, and reduced by KLF8 knockdown, we hypothesized that KLF8 may regulate expression of components of the pluripotency network that are associated with breast cancer stem cells. To test this idea, we overexpressed or reduced KLF8 expression in MDA-MB-231 and SUM159 cells and assessed mRNA and protein expression of major stem cell factors that are associated with breast tumor development and include OCT4, SOX2, NANOG and c-MYC (33–35). KLF8 overexpression in MDA-MB-231 (**Figure 3A**) and SUM159 (**Supplemental Figure 2A**) cells significantly increased protein levels of stem cell factors OCT4, SOX2, NANOG and c-MYC and significantly increased RNA levels of stem cell factors OCT4, SOX2 and NANOG in MDA-MB-231 (**Figure 3B**) and SUM159 cells (**Supplemental Figure 2B**). Conversely, KLF8 knockdown in MDA-MB-231 (**Figure 3C**) and SUM159 (**Supplemental Figure 2C**) cells significantly reduced protein levels and mRNA levels of OCT4, SOX2, NANOG and c-MYC in MDA-MB-231 (**Figure 3D**) and SUM159 (**Supplemental Figure 2D**) cells compared to controls. Together, these data suggested that KLF8 regulates RNA and protein levels of the pluripotency transcription factors in triple negative breast cancer cells.

### KLF8 regulates chemoresistance in breast cancer cells

**Figure 4:**
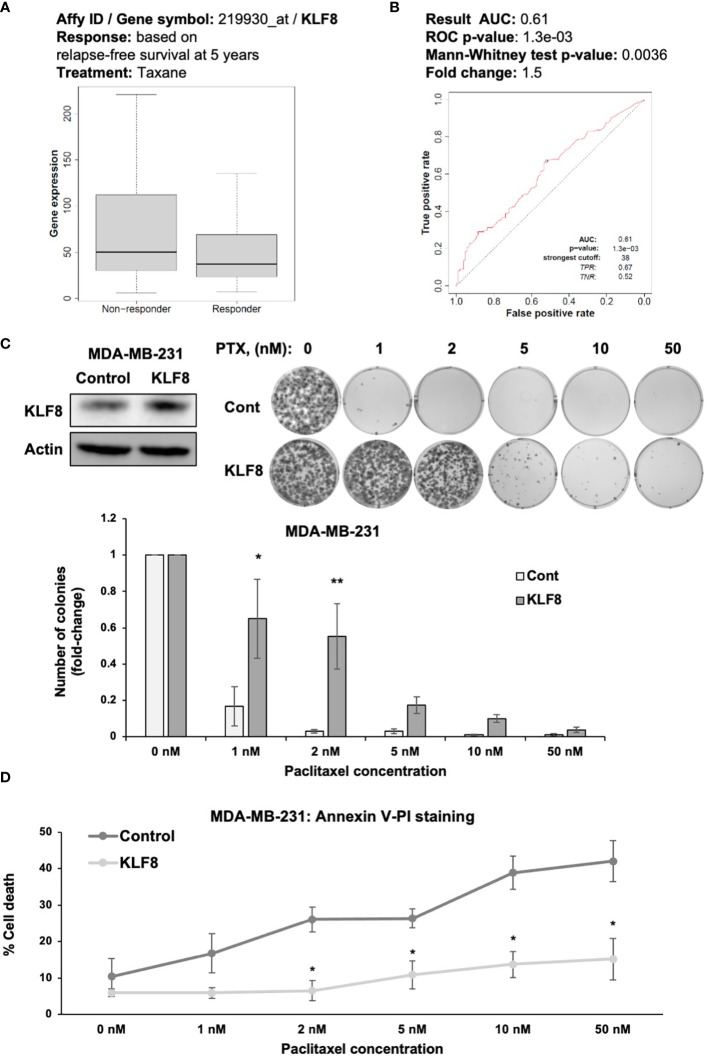
KLF8 promotes resistance to paclitaxel in breast cancer cells *in vitro.* **(A)** Data-based analysis showing KLF8 expression in breast cancer patients with differential response to taxane treatment. **(B)** Data-based analysis showing ROC-plot of KLF8 expression in different groups of breast cancer patients treated with taxane. **(C)** Lysates of MDA-MB-231 cells stably overexpressing control or with KLF8 were collected for immunoblot using indicated antibodies (top-left). MDA-MB-231 cells stably overexpressing control or KLF8, treated with increasing dose of paclitaxel for 48h, were grown in clonogenic assay for 10-14 days. Colonies were stained, counted. Representative images show stained colonies formed in the clonogenic assay after 10-14 days from MDA-MB-231 cells overexpressing control or KLF8, treated with increasing dose of paclitaxel for 48h (top-right). Quantified graph of counted colonies after 10-14 days from MDA-MB-231 cells overexpressing control or KLF8, treated with increasing dose of paclitaxel for 48h, in clonogenic assay (bottom). **(D)** Quantified graph showing fraction of apoptotic cells from MDA-MB-231 cells overexpressing control or KLF8, treated with increasing dose of paclitaxel for 48h. Two-way ANOVA with Holm-Sidak test reported as mean ± SEM, *p<0.05, and **p<0.01.

An important characteristic of CSCs is the ability of avoiding cells death induced by chemotherapeutic agents (36). Therefore, we hypothesized that KLF8 may also promote resistance to chemotherapy in breast cancer cells (37). We examined whether KLF8 expression correlated with patients’ response to chemotherapy, we used ROC plotter (28) to assess the potential correlation between KLF8 expression and patient response to taxane, a first-line treatment for treating breast cancer patients (28). In TNBC patients we found that KLF8 levels are higher in non-responders to taxane compared to patients that responded to taxane (**Figure 4A**). Moreover, the area under the curve was greater than 0.6 (28), indicating that KLF8 may be a marker for taxane resistance in breast cancer patients (**Figure 4B**). To test whether KLF8 can regulate chemoresistance in breast cancer cells, we overexpressed KLF8 in MDA-MB-231 and SUM159 cells, followed by treatment with increasing doses of paclitaxel for 48h. Cells survival were then assessed by clonogenic growth and Annexin-V/Propidium Iodide staining. Paclitaxel treatment reduced the number of colonies in a dose-dependent manner in both MDA-MB-231 (**Figure 4C**) and SUM159 (**Supplemental Figure 3A**) cells. Importantly, the number of colonies formed in KLF8 overexpressing MDA-MB-231 (**Figure 4C**) and SUM159 (**Supplemental Figure 3A**) cells was significantly higher compared to control cells. Consistent with this data, the fraction of apoptotic cells induced by paclitaxel was significantly reduced in KLF8 overexpression cells compared to control cells (**Figure 4D**). In addition, we tested whether KLF8 overexpression increased mammosphere formation in paclitaxel treated cells compared to control cells. We detect a significant increase in KLF8 overexpressing cells in mammosphere formation efficiency in paclitaxel treated mammospheres compared to control cells (**Supplemental Figure 3B**). We also detect increased expression of CSC factors in KLF8 overexpressing cells treated with PTX compared to controls (**Supplemental Figure 3C**). Together, these data suggest an important role of KLF8 in promoting resistance against paclitaxel treatment, and KLF8 may serve as predictive marker for taxane resistance in TNBC patients.

### KLF8 regulates OGT and forms feedback loop to regulate CSCs

**Figure 5:**
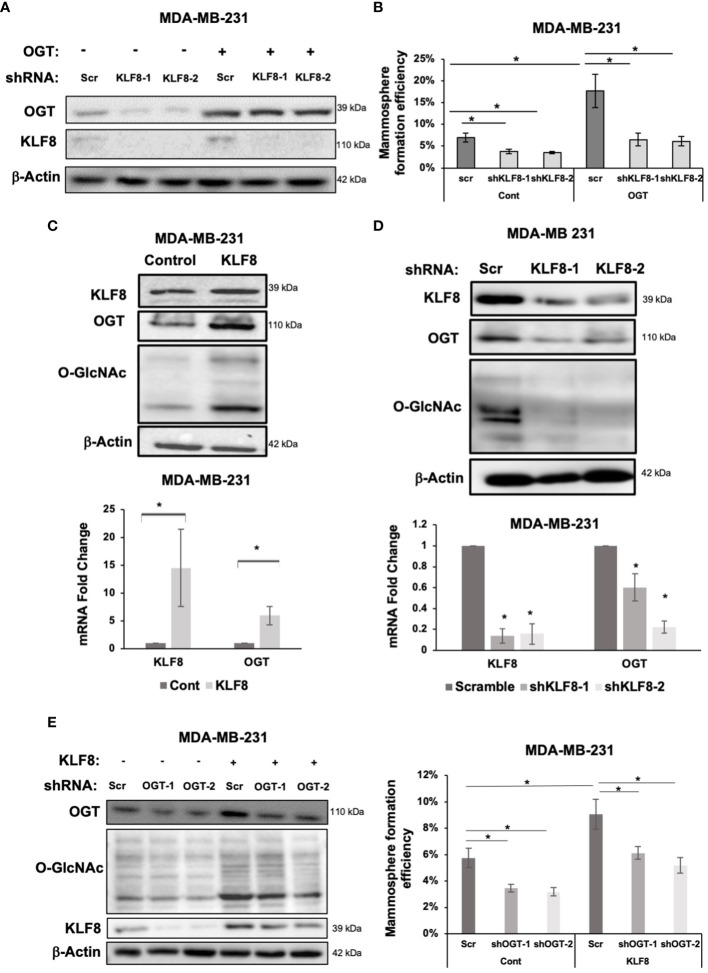
KLF8 and OGT/O-GlcNAc formed a potential feed-forward loop in breast cancer cells. **(A)** Lysates of MDA-MB-231 cells overexpressing control or OGT, transduced with control or KLF8 shRNA, were collected for immunoblot analysis using antibodies against indicated targets **(B)** Quantified graph showing mammosphere formation efficiency of MDA-MB-231 cells overexpressing control or OGT, transduced with control or KLF8 shRNA, after 5-7 days. **(C)** Lysates of MDA-MB-231 cells overexpressing control or OGT were collected for immunoblot analysis using indicated antibodies (top). Quantified graph showing relative mRNA level, detected by qRT-PCR, of KLF8 and OGT from MDA-MB-231 cells overexpressing control or OGT in mammosphere formation assay. **(D)** Lysates of MDA-MB-231 cells stably expressing control or KLF8 shRNA were collected for immunoblot analysis using indicated antibodies (top). Quantified graph showing relative mRNA level, detected by qRT-PCR, of KLF8 and OGT from MDA-MB-231 cells stably expressing control or KLF8 shRNA. **(E)** Lysates of MDA-MB-231 cells stably overexpressing control or KLF8, transduced with control of OGT shRNA, were collected for immunoblot analysis using indicated antibodies. **(B)** Quantified graph showing mammosphere formation efficiency of MDA-MB-231 cells overexpressing control or KLF8, transduced with control of OGT shRNA, after 5-7 days in mammosphere formation assay. Student *t* test reported as mean ± SEM, *p<0.05.

A previous study identified a critical role of OGT in promoting CSCs phenotype, and tumor initiation, potentially *via* upregulating cancer stem cell factors and upregulation of KLF8 levels (18). To test whether KLF8 is a downstream effector of OGT in CSCs, we stably overexpressed OGT in MDA-MB-231 cells and reduced KLF8 expression using shRNA (**Figure 5A**). As previously reported, overexpressing OGT significantly increased mammosphere formation efficiency in MDA-MB-231 cells (**Figure 5B**). However, knockdown of KLF8 in OGT overexpressing cells reduced mammosphere formation back to the basal level (**Figure 5B**). Immunoblot analysis of MDA-MB-231 cells overexpressing KLF8 (**Figure 5C**) showed both OGT and O-GlcNAc was elevated suggesting a potential feedback loop between KLF8 and OGT in breast cancer cells. Indeed, we found cells overexpressing KLF8 had significant increase in OGT protein levels and O-GlcNAc compared to controls (**Figure 5C**; **Supplemental Figure 4A**). Since KLF8 is a transcription factor, we hypothesized that KLF8 regulates OGT at the mRNA level. To test this idea, we assessed OGT mRNA levels using qRT-PCR in MDA-MB-231 cells overexpressing KLF8. Increased KFL8 expression led to significant increase in OGT mRNA level (**Figure 5C**). Conversely, we found in KLF8 knockdown MDA-MB-231 cells contained reduced OGT and O-GlcNAc protein levels (**Figure 5D**; **Supplemental Figure 4B**) and significant reduction of OGT mRNA (**Figure 5D**) levels compared to controls. To confirm that OGT and KLF8 co-regulate each other to promote stem cell properties in breast cancer, we overexpressed KLF8 in TNBC cells and transduced the cells with either control or shRNA targeting OGT, followed by assessing mammosphere formation efficiency. As expected, overexpression of KLF8 increased OGT and O-GlcNAc levels (**Figure 5E**) in breast cancer cells. Knockdown of OGT in MDA-MB-231 cells significantly reduced mammosphere formation, while KLF8 overexpression increased the mammosphere formation efficiency. Importantly, knockdown of OGT in KLF8 overexpression reduced mammosphere formation in MDA-MB-231 cells (**Figure 5E**), suggesting both OGT and KLF8 are needed to promote mammosphere formation and stem cell properties of breast cancer cells.

### *KLF8* regulates breast cancer tumor growth *in vivo*

**Figure 6:**
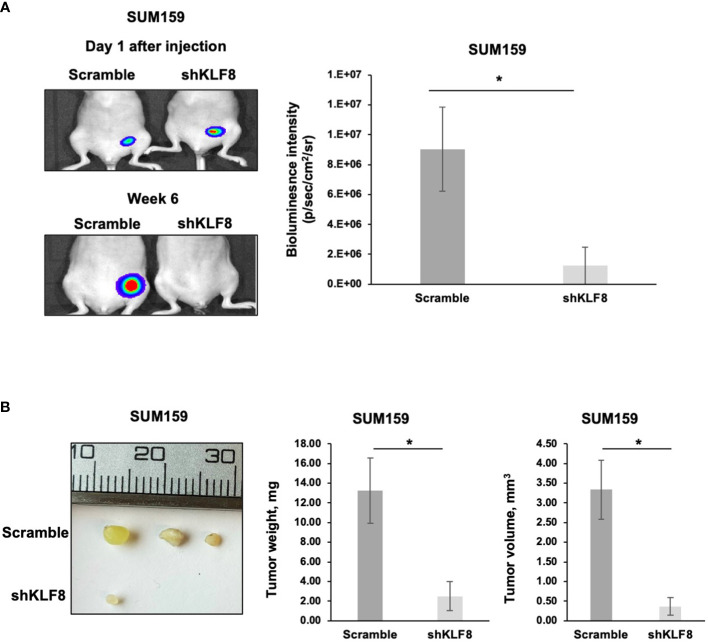
Reduced KLF8 expression impaired breast tumor growth *in vivo*. **(A)** Representative images of tumors in immune-compromised mice, formed from MDA-MB-231 cells stably expressing luciferase, transduced with control or KLF8 shRNA 1 day and 6 weeks after injection (left). Quantified graph showing bioluminescence signal from tumors formed from MDA-MB-231 cells stably expressing luciferase, transduced with control (n=4) or KLF8 (n=4) shRNA, 6 weeks after injection (right). **(B)** Representative images of tumors formed from MDA-MB-231 cells stably expressing luciferase, transduced with control or KLF8 shRNA, taken from mammary fat pad of immune-compromised mice 6 weeks after injection (left). Quantified graph showing weight of tumors formed from MDA-MB-231 cells stably expressing luciferase, transduced with control (n=4) or KLF8 (n=4) shRNA (middle). Quantified graph showing volume of tumors formed from TNBC cells MDA-MB-231 stably expressing luciferase, transduced with control (n=4) or KLF8 (n=4) shRNA (right). Student *t* test reported as mean ± SEM, *p<0.05.

A key characteristic of CSCs is the ability to promote and maintain long-term tumor growth. To test whether KLF8 is required for breast tumor growth, we injected luciferase expressing SUM159 cells stably expressing control or KLF8 shRNA into the mammary fat pad of immunodeficient mice. At the end point, tumors were surgically removed, and tumor weight and volume were assessed. Knockdown of KLF8 in SUM159 cell significantly reduced bioluminescent signal (**Figure 6A**), tumor volume (**Figure 6B**) and tumor weight (**Figure 6B**) compared to controls. Consistent with this data, poor outcome of all breast cancer patients (**Supplemental Figure 5A**) and basal-type breast cancer patients (**Supplemental Figure 5B**) is associated with high expression of KLF8 RNA. Thus, our data suggests that KFL8 plays a key role in regulating CSCs phenotypes *in vitro*, forms a feed-forward regulation with OGT, regulates triple negative breast tumor growth *in vivo* and associates with poor clinical outcome in breast cancer patients.

## Discussion

Breast cancer remains a major burden for woman worldwide, ranking as the second-leading cause of cancer-related death (1). Poor outcome of breast cancer usually associates with metastasis, drug resistance and tumor relapse. All these processes are proposed to be regulated by a small population of CSCs (5, 7, 8). By targeting CSCs in combination with conventional chemotherapy, efficiency of breast cancer treatment may be improved (38, 39). Therefore, it is important to understand regulation mechanism of CSCs to identify potential target for CSCs specific treatment. Our previous study identified OGT and O-GlcNAc as a key driver of tumor initiation and stemness of breast cancer cells (18). We also identified KLF8 as a potential target of OGT in regulating CSCs. Here, we showed that KLF8 is necessary and sufficient to promote mammosphere formation and CSCs population in TNBC cells. Importantly, we also confirmed the role of KLF8 downstream of OGT in TNBC. Interestingly, KLF8 and OGT work together and form a feed-forward loop to regulate CSCs. KLF8 also regulates expression of major stem cell markers at both protein and mRNA level in TNBC cells. Importantly, overexpression of KLF8 induced resistance against paclitaxel *in vitro* suggesting a potential role of KLF8 in regulating chemoresistance in triple negative breast cancer cells.

KLF8 is one member of the Kruppel-like transcription (KLF) factor family, which is involved in a wide range of cellular activity. Many members of KLF family are involved in regulating proliferation, self-renewal, and differentiation. KLF2, KLF4 and KLF5 were reported to be essential to maintain the pluripotent state of embryonic stem cells (40). Importantly, high expression of KLF members associates with poor survival of patients with cancer (41–43). Similarly, poor outcome of breast cancer also associates with high mRNA level of KLF8 (**Supplemental Figure 3**), suggesting a potential role of KLF8 in the breast tumor development. Indeed, KLF8 directly represses expression of E-Cadherin, a key epithelial cell marker, inducing epithelial-mesenchymal transition (20). In breast cancer cells, KLF8 also induces expression of MMP9, an enzyme critical for breaking through the extracellular matrix during cancer cell invasion and migration (24). Altogether, KLF8 is believed to play an important role during metastasis. In this study, we showed that KLF8 knockdown in TNBC cells significantly reduced mammosphere formation and BCSCs population, while overexpression of KLF8 increased the mammosphere formation capacity and BCSCs population. We also provide evidence that KLF8 can regulate expression of key stem cell factors, including OCT4, SOX2, c-MYC and NANOG, at both protein and mRNA level. By upregulating expression of these stem cell factors, KLF8 promotes the acquisition of stem-like cell phenotype in breast cancer cells. KLF8 has also been shown to regulate breast cancer proliferation (44) thus it’s possible that KLF8 regulates proliferation of both non-cancer stem cells and cancer stem cells. Our results show a potential role of KLF8 in regulating CSCs in breast cancer cells.

One notable characteristics of CSCs is the resistance to chemotherapy. High levels of KLF8 expression were associated with breast cancer patients not-responding to taxanes compared to patients that responded (**Figure 4A**). This data along with the ROC plot analysis (**Figure 4B**) suggests KLF8 may serve as a predictive marker for chemoresistance in breast cancer. Consistent with the database analysis, overexpression of KLF8 in TNBC cells significantly reduced paclitaxel-induced apoptosis and increased resistance to the drug (**Figure 4**). These results identify KLF8 as a potential driver of chemoresistance in breast cancer cells, which may serve as a potential predictive marker for chemotherapy outcome in breast cancer.

KLF8 was found to be among the most upregulated genes caused by OGT overexpression in breast cancer cells, suggesting a potential regulation of KLF8 by OGT. The regulation was confirmed by increased KLF8 level in TNBC cells with OGT overexpression (18). Depletion of KLF8 was able to abolish the effect of OGT overexpression in regulating mammosphere formation in TNBC cells, confirming the role of KLF8 downstream of OGT in breast cancer cells. Interestingly, our study also showed a co-regulation between OGT and KLF8, as KLF8 regulates OGT RNA and protein levels and KLF8 requires OGT for mammosphere formation, suggesting a potential feed-forward loop of OGT and KLF8 to regulate CSCs in breast cancer. OGT expression has been shown to be upregulated by c-MYC in breast cancer cells (45). Our study showed a positive regulation of c-MYC by KLF8, suggesting a possible regulation of OGT by KLF8 *via* upregulation of c-MYC. Since cancer stem cells are associated with increases metastasis and both OGT (46) and KLF8 (24) have been linked to regulation of metastasis in breast cancer, it will be of interest to study whether the OGT and KLF8 feed forward loop plays a role in metastasis. Together, these data confirm the role of KLF8 and the KLF8/OGT loop in promoting stemness and chemoresistance of triple negative breast cancer cell, which may be used to develop strategy in BCSCs-specific treatment.

## Data availability statement

The original contributions presented in the study are included in the article/**Supplementary Material**. Further inquiries can be directed to the corresponding authors.

## Ethics statement

The animal study was reviewed and approved by Institutional Animal Care and Use Committee.

## Author contributions

GL performed most of the experimental work. EE, TD, JM and ML helped with experimental work. GL and MR participated in study conception. GL, LB and MR designed experiments and performed data analysis and interpretation. GL and MR drafted the manuscript. GL, EE and MR made edits. All authors contributed to the article and approved the submitted version.

## Supplementary material

Supplementary Figure 1 Reduced KLF8 expression impaired mammosphere formation of TNBC cells. **(A)** Lysates from SUM159 cells stably expressing control or KLF8 shRNA was collected for immunoblot analysis using indicated antibodies (top-left). SUM159 cells stably expressing control or KLF8 shRNA were grown in mammosphere formation assay for 5-7 days. Representative images of mammosphere were taken (top-right) (scale bar 400 μm), and mammosphere larger than 50µm were counted and mammosphere formation efficiency was quantified. Quantified graph showing primary mammosphere formation efficiency of SUM159 cells stably expressing control or KLF8 shRNA. Primary mammosphere were collected and culture again in mammosphere culture condition for 5-7 days to form secondary mammosphere (bottom). **(B)** Lysates from PDX cells HCI-10 stably expressing control or KLF8 shRNA was collected for immunoblot analysis using indicated antibodies (top-left). HCI-10 cells stably expressing control or KLF8 shRNA were grown in mammosphere formation assay for 5-7 days. Representative images of mammosphere were taken (top-right) (scale bar 400 μm), and mammosphere larger than 50µm were counted and mammosphere formation efficiency was quantified. Quantified graph showing primary mammosphere formation efficiency of HCI-10 cells stably expressing control or KLF8 shRNA. Primary mammosphere were collected and cultured again in mammosphere culture condition for 5- days to form secondary mammosphere (bottom). (Student *t* test reported as mean ± SEM, *p<0.05, and **p<0.01.

Supplementary Figure 2 KLF8 regulates expression of stem cell markers in breast cancer cells. **(A)** Lysates from SUM159 cells stably overexpressing control or KLF8 were collected for immunoblot analysis of CSCs markers using indicated antibodies (left), and quantified graph of relative level of CSCs detected by immunoblot from SUM159 cells control or with KLF8 overexpression (right). **(B)** Quantified graph showing relative mRNA level of CSCs markers as detected by qRT-PCR using probes against genes in SUM159 cells stably overexpressing control or KLF8. **(C)** Lysates from SUM159 cells stably expressing control or KLF8 shRNA were collected for immunoblot analysis of CSCs markers using indicated antibodies (left), and quantified graph of relative level of CSCs levels (right). **(D)** Quantified graph showing relative mRNA level of CSCs markers as detected by qRT-PCR using probes against indicated targets in SUM159 cells stably expressing control or KLF8 shRNA. Student *t* test reported as mean ± SEM, *p<0.05.

Supplementary Figure 3 KLF8 promotes resistance to paclitaxel in breast cancer cells *in vitro.* **(A)** Lysates of SUM159 cells overexpressing control or KLF8 were collected for immunoblot using indicated antibodies (top-left). SUM159 cells overexpressing control or KLF8, treated with increasing dose of paclitaxel for 48h, were grown in clonogenic assay for 10-14 days. Colonies were stained, counted. Representative images show stained colonies formed in the clonogenic assay after 10-14 days from SUM159 cells control or with KLF8 overexpression, treated with increasing dose of paclitaxel for 48h (top-right). Quantified graph of counted colonies after 10-14 days from SUM159 cells control or with KLF8 overexpression, treated with increasing dose of paclitaxel for 48h, in clonogenic assay (bottom). Two-way ANOVA with Holm-Sidak test reported as mean ± SEM, **p<0.01. ***p<0.001. **(B)** MDA-MB-231 cells control or stably overexpressing KLF8 were treated with 2nM final concentration of Paclitaxel for 48h and cells were cultured in mammosphere formation assay for 5-7 days. Mammosphere larger than 50 µm were counted. **(C)** MDA-MB-231 cells control or stably overexpressing KLF8 were treated with 2nM final concentration of Paclitaxel for 48h and cell lysates were collected for immunoblot analysis using indicated antibodies.

Supplementary Figure 4
**(A)** Quantified graph showing relative protein level of KLF8, OGT, and O-GlcNAc in MDA-MB-231 cells control or overexpressing KLF8 as detected by immunoblot using indicated antibodies (corresponding to ). Student *t* test reported as mean ± SEM, *p<0.05. **(B)** Quantified graph showing relative protein level of KLF8, OGT, and O-GlcNAc in MDA-MB-231 cells expressing control shRNA or KLF8 shRNA as detected by immunoblot using indicated antibodies (corresponding to ). Student *t* test reported as mean ± SEM, *p<0.05.

Supplementary Figure 5 Increased KLF8 expression associates with poor outcome of breast cancer. **(A)** Kaplan-Meier plot showing overall survival of all breast cancer patients with different level of KLF8 mRNA. **(B)** Kaplan-Meier plot showing overall survival of basal-type breast cancer patients with different level of KLF8 mRNA.

## References

[1] Ferlay J Colombet M Soerjomataram I Parkin DM Pineros M Znaor A. Cancer statistics for the year 2020: An overview. Int J Cancer (2021) 149(4):778–89. doi: 10.1002/ijc.33588 33818764

[2] Yersal O Barutca S. Biological subtypes of breast cancer: Prognostic and therapeutic implications. World J Clin Oncol (2014) 5(3):412–24. doi: 10.5306/wjco.v5.i3.412 PMC4127612 25114856

[3] Foulkes WD Smith IE Reis-Filho JS. Triple-negative breast cancer. N Engl J Med (2010) 363(20):1938–48. doi: 10.1056/NEJMra1001389 21067385

[4] Bai X Ni J Beretov J Graham P Li Y. Triple-negative breast cancer therapeutic resistance: Where is the achilles' heel? Cancer Lett (2021) 497:100–11. doi: 10.1016/j.canlet.2020.10.016 33069769

[5] He L Wick N Germans SK Peng Y. The role of breast cancer stem cells in chemoresistance and metastasis in triple-negative breast cancer. Cancers (Basel) (2021) 13(24):6209. doi: 10.3390/cancers13246209 34944829 PMC8699562

[6] Das B Pal B Bhuyan R Li H Sarma A Gayan S. MYC regulates the HIF2alpha stemness pathway *via* nanog and Sox2 to maintain self-renewal in cancer stem cells versus non-stem cancer cells. Cancer Res (2019) 79(16):4015–25. doi: 10.1158/0008-5472.CAN-18-2847 PMC6701948 31266772

[7] Batlle E Clevers H. Cancer stem cells revisited. Nat Med (2017) 23(10):1124–34. doi: 10.1038/nm.4409 28985214

[8] De Angelis ML Francescangeli F Zeuner A. Breast cancer stem cells as drivers of tumor chemoresistance, dormancy and relapse: New challenges and therapeutic opportunities. Cancers (Basel) (2019) 11(10):1569. doi: 10.3390/cancers11101569 31619007 PMC6826533

[9] Ferrer CM Sodi VL Reginato MJ. O-GlcNAcylation in cancer biology: Linking metabolism and signaling. J Mol Biol (2016) 428(16):3282–94. doi: 10.1016/j.jmb.2016.05.028 PMC4983259 27343361

[10] Itkonen HM Loda M Mills IG. O-GlcNAc transferase - an auxiliary factor or a full-blown oncogene? Mol Cancer Res (2021) 19(4):555–64. doi: 10.1158/1541-7786.MCR-20-0926 33472950

[11] Ma J Wu C Hart GW. Analytical and biochemical perspectives of protein O-GlcNAcylation. Chem Rev (2021) 121(3):1513–81. doi: 10.1021/acs.chemrev.0c00884 33416322

[12] Ciraku L Esquea EM Reginato MJ. O-GlcNAcylation regulation of cellular signaling in cancer. Cell Signal (2022) 90:110201. doi: 10.1016/j.cellsig.2021.110201 34800629 PMC8712408

[13] Shimizu M Tanaka N. IL-8-induced O-GlcNAc modification *via* GLUT3 and GFAT regulates cancer stem cell-like properties in colon and lung cancer cells. Oncogene (2019) 38(9):1520–33. doi: 10.1038/s41388-018-0533-4 30305725

[14] Fuentes-Garcia G Castaneda-Patlan MC Vercoutter-Edouart AS Lefebvre T Robles-Flores M. O-GlcNAcylation is involved in the regulation of stem cell markers expression in colon cancer cells. Front Endocrinol (Lausanne). (2019) 10:289. doi: 10.3389/fendo.2019.00289 31139149 PMC6518200

[15] Shimizu M Shibuya H Tanaka N. Enhanced O-GlcNAc modification induced by the RAS/MAPK/CDK1 pathway is required for SOX2 protein expression and generation of cancer stem cells. Sci Rep (2022) 12(1):2910. doi: 10.1038/s41598-022-06916-y 35190631 PMC8861017

[16] Cao B Duan M Xing Y Liu C Yang F Li Y. O-GlcNAc transferase activates stem-like cell potential in hepatocarcinoma through O-GlcNAcylation of eukaryotic initiation factor 4E. J Cell Mol Med (2019) 23(4):2384–98. doi: 10.1111/jcmm.14043 PMC6433694 30677218

[17] Yuan Y Wang L Ge D Tan L Cao B Fan H. Exosomal O-GlcNAc transferase from esophageal carcinoma stem cell promotes cancer immunosuppression through up-regulation of PD-1 in CD8(+) T cells. Cancer Lett (2021) 500:98–106. doi: 10.1016/j.canlet.2020.12.012 33307156

[18] Akella NM Le Minh G Ciraku L Mukherjee A Bacigalupa ZA Mukhopadhyay D. O-GlcNAc transferase regulates cancer stem-like potential of breast cancer cells. Mol Cancer Res (2020) 18(4):585–98. doi: 10.1158/1541-7786.MCR-19-0732 PMC7127962 31974291

[19] Wang X Zhao J. KLF8 transcription factor participates in oncogenic transformation. Oncogene (2007) 26(3):456–61. doi: 10.1038/sj.onc.1209796 16832343

[20] Wang X Zheng M Liu G Xia W McKeown-Longo PJ Hung MC. Kruppel-like factor 8 induces epithelial to mesenchymal transition and epithelial cell invasion. Cancer Res (2007) 67(15):7184–93. doi: 10.1158/0008-5472.CAN-06-4729 17671186

[21] Zhang H Liu L Wang Y Zhao G Xie R Liu C. KLF8 involves in TGF-beta-induced EMT and promotes invasion and migration in gastric cancer cells. J Cancer Res Clin Oncol (2013) 139(6):1033–42. doi: 10.1007/s00432-012-1363-3 PMC11824695 23504025

[22] Yan Q Zhang W Wu Y Wu M Zhang M Shi X. KLF8 promotes tumorigenesis, invasion and metastasis of colorectal cancer cells by transcriptional activation of FHL2. Oncotarget (2015) 6(28):25402–17. doi: 10.18632/oncotarget.4517 PMC4694840 26320172

[23] Lu H Hu L Yu L Wang X Urvalek AM Li T. KLF8 and FAK cooperatively enrich the active MMP14 on the cell surface required for the metastatic progression of breast cancer. Oncogene (2014) 33(22):2909–17. doi: 10.1038/onc.2013.247 PMC3929536 23812425

[24] Wang X Lu H Urvalek AM Li T Yu L Lamar J. KLF8 promotes human breast cancer cell invasion and metastasis by transcriptional activation of MMP9. Oncogene (2011) 30(16):1901–11. doi: 10.1038/onc.2010.563 PMC3952074 21151179

[25] Caldwell SA Jackson SR Shahriari KS Lynch TP Sethi G Walker S. Nutrient sensor O-GlcNAc transferase regulates breast cancer tumorigenesis through targeting of the oncogenic transcription factor FoxM1. Oncogene (2010) 29(19):2831–42. doi: 10.1038/onc.2010.41 20190804

[26] Ferrer CM Lynch TP Sodi VL Falcone JN Schwab LP Peacock DL. O-GlcNAcylation regulates cancer metabolism and survival stress signaling *via* regulation of the HIF-1 pathway. Mol Cell (2014) 54(5):820–31. doi: 10.1016/j.molcel.2014.04.026 PMC4104413 24857547

[27] Gyorffy B. Survival analysis across the entire transcriptome identifies biomarkers with the highest prognostic power in breast cancer. Comput Struct Biotechnol J (2021) 19:4101–9. doi: 10.1016/j.csbj.2021.07.014 PMC8339292 34527184

[28] Fekete JT Gyorffy B. ROCplot.org: Validating predictive biomarkers of chemotherapy/hormonal therapy/anti-HER2 therapy using transcriptomic data of 3,104 breast cancer patients. Int J Cancer (2019) 145(11):3140–51. doi: 10.1002/ijc.32369 31020993

[29] Grimshaw MJ Cooper L Papazisis K Coleman JA Bohnenkamp HR Chiapero-Stanke L. Mammosphere culture of metastatic breast cancer cells enriches for tumorigenic breast cancer cells. Breast Cancer Res (2008) 10(3):R52. doi: 10.1186/bcr2106 18541018 PMC2481500

[30] Fatima I El-Ayachi I Taotao L Lillo MA Krutilina RI Seagroves TN. The natural compound jatrophone interferes with wnt/beta-catenin signaling and inhibits proliferation and EMT in human triple-negative breast cancer. PloS One (2017) 12(12):e0189864. doi: 10.1371/journal.pone.0189864 29281678 PMC5744972

[31] Marcato P Dean CA Giacomantonio CA Lee PW. Aldehyde dehydrogenase: its role as a cancer stem cell marker comes down to the specific isoform. Cell Cycle (2011) 10(9):1378–84. doi: 10.4161/cc.10.9.15486 21552008

[32] Thiagarajan PS Hitomi M Hale JS Alvarado AG Otvos B Sinyuk M. Development of a fluorescent reporter system to delineate cancer stem cells in triple-negative breast cancer. Stem Cells (2015) 33(7):2114–25. doi: 10.1002/stem.2021 PMC4494654 25827713

[33] Leis O Eguiara A Lopez-Arribillaga E Alberdi MJ Hernandez-Garcia S Elorriaga K. Sox2 expression in breast tumours and activation in breast cancer stem cells. Oncogene (2012) 31(11):1354–65. doi: 10.1038/onc.2011.338 21822303

[34] Wang D Lu P Zhang H Luo M Zhang X Wei X. Oct-4 and nanog promote the epithelial-mesenchymal transition of breast cancer stem cells and are associated with poor prognosis in breast cancer patients. Oncotarget (2014) 5(21):10803–15. doi: 10.18632/oncotarget.2506 PMC4279411 25301732

[35] Lee HY Cha J Kim SK Park JH Song KH Kim P. C-MYC drives breast cancer metastasis to the brain, but promotes synthetic lethality with TRAIL. Mol Cancer Res (2019) 17(2):544–54. doi: 10.1158/1541-7786.MCR-18-0630 30266755

[36] Abdullah LN Chow EK. Mechanisms of chemoresistance in cancer stem cells. Clin Transl Med (2013) 2(1):3. doi: 10.1186/2001-1326-2-3 23369605 PMC3565873

[37] Yuan H Guo H Luan X He M Li F Burnett J. Albumin nanoparticle of paclitaxel (Abraxane) decreases while taxol increases breast cancer stem cells in treatment of triple negative breast cancer. Mol Pharm (2020) 17(7):2275–86. doi: 10.1021/acs.molpharmaceut.9b01221 PMC8829889 32485107

[38] Phi LTH Sari IN Yang YG Lee SH Jun N Kim KS. Cancer stem cells (CSCs) in drug resistance and their therapeutic implications in cancer treatment. Stem Cells Int (2018) 2018:5416923. doi: 10.1155/2018/5416923 29681949 PMC5850899

[39] Yang L Shi P Zhao G Xu J Peng W Zhang J. Targeting cancer stem cell pathways for cancer therapy. Signal Transduct Target Ther (2020) 5(1):8. doi: 10.1038/s41392-020-0110-5 32296030 PMC7005297

[40] Jiang J Chan YS Loh YH Cai J Tong GQ Lim CA. A core klf circuitry regulates self-renewal of embryonic stem cells. Nat Cell Biol (2008) 10(3):353–60. doi: 10.1038/ncb1698 18264089

[41] Kim SH Park YY Cho SN Margalit O Wang D DuBois RN. Kruppel-like factor 12 promotes colorectal cancer growth through early growth response protein 1. PloS One (2016) 11(7):e0159899. doi: 10.1371/journal.pone.0159899 27442508 PMC4956169

[42] Lyu J Wang J Miao Y Xu T Zhao W Bao T. KLF7 is associated with poor prognosis and regulates migration and adhesion in tongue cancer. Oral Dis (2022) 28(3):577–84. doi: 10.1111/odi.13767 33393169

[43] Wang H Shi Y Chen CH Wen Y Zhou Z Yang C. KLF5-induced lncRNA IGFL2-AS1 promotes basal-like breast cancer cell growth and survival by upregulating the expression of IGFL1. Cancer Lett (2021) 515:49–62. doi: 10.1016/j.canlet.2021.04.016 34052325

[44] Li T Lu H Mukherjee D Lahiri SK Shen C Yu L. Identification of epidermal growth factor receptor and its inhibitory microRNA141 as novel targets of kruppel-like factor 8 in breast cancer. Oncotarget (2015) 6(25):21428–42. doi: 10.18632/oncotarget.4077 PMC4673276 26025929

[45] Sodi VL Khaku S Krutilina R Schwab LP Vocadlo DJ Seagroves TN. mTOR/MYC axis regulates O-GlcNAc transferase expression and O-GlcNAcylation in breast cancer. Mol Cancer Res (2015) 13(5):923–33. doi: 10.1158/1541-7786.MCR-14-0536 PMC4433402 25636967

[46] Ferrer CM Lu TY Bacigalupa ZA Katsetos CD Sinclair DA Reginato MJ. O-GlcNAcylation regulates breast cancer metastasis *via* SIRT1 modulation of FOXM1 pathway. Oncogene (2017) 36(4):559–69. doi: 10.1038/onc.2016.228 PMC5192006 27345396

