# Supplementary figures and images for: Kruppel-like factor 8 regulates triple negative breast cancer stem cell-like activity
Source: Front Oncol. 2023 Apr 19;13:1141834. doi: 10.3389/fonc.2023.1141834 (PMC10155275; doi:10.3389/fonc.2023.1141834)

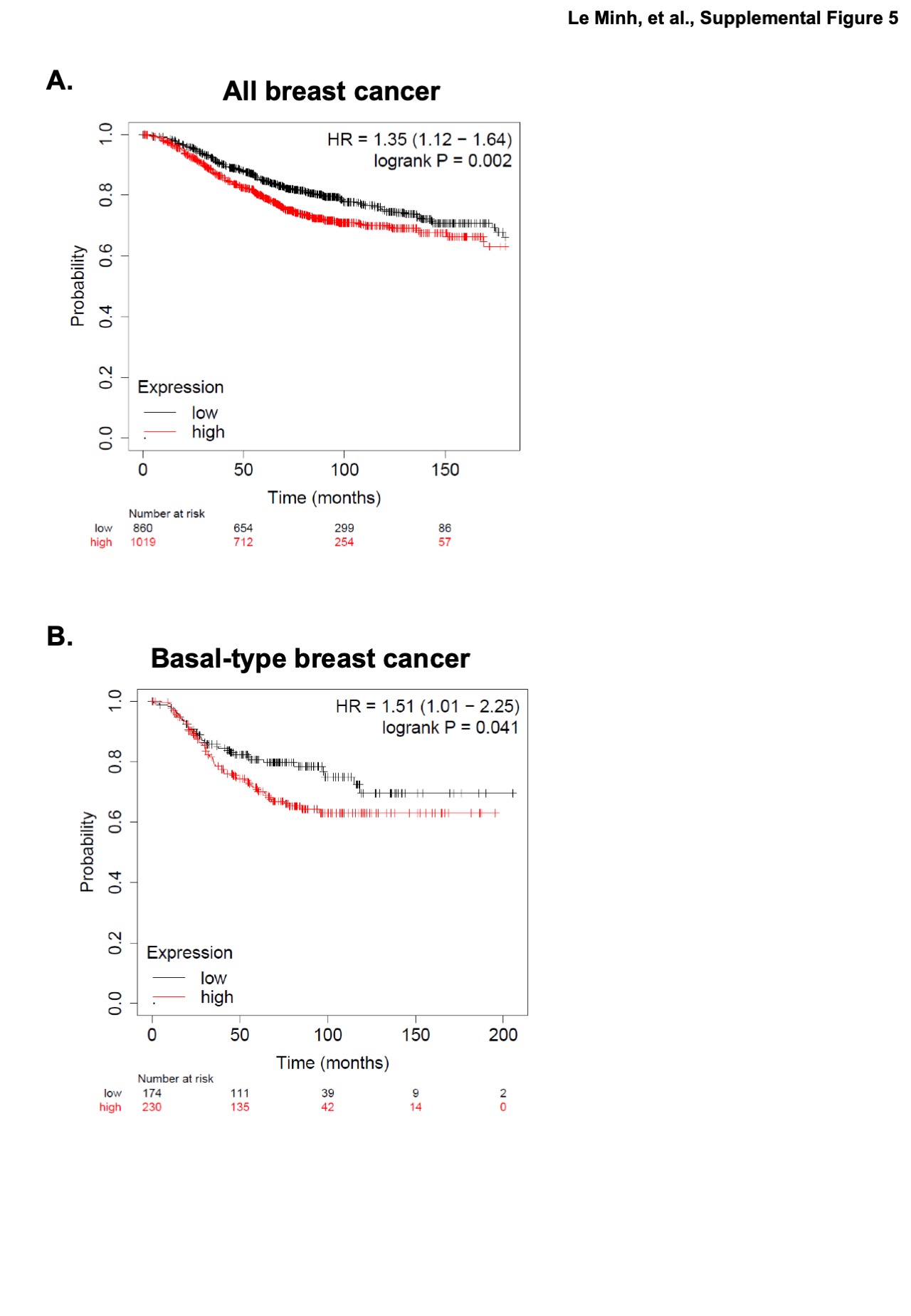

Supplement: Supplementary Figure 1 — Reduced KLF8 expression impaired mammosphere formation of TNBC cells. (A) Lysates from SUM159 cells stably expressing control or KLF8 shRNA was collected for immunoblot analysis using indicated antibodies (top-left). SUM159 cells stably expressing control or KLF8 shRNA were grown in mammosphere formation assay for 5-7 days. Representative images of mammosphere were taken (top-right) (scale bar 400 μm), and mammosphere larger than 50µm were counted and mammosphere formation efficiency was quantified. Quantified graph showing primary mammosphere formation efficiency of SUM159 cells stably expressing control or KLF8 shRNA. Primary mammosphere were collected and culture again in mammosphere culture condition for 5-7 days to form secondary mammosphere (bottom). (B) Lysates from PDX cells HCI-10 stably expressing control or KLF8 shRNA was collected for immunoblot analysis using indicated antibodies (top-left). HCI-10 cells stably expressing control or KLF8 shRNA were grown in mammosphere formation assay for 5-7 days. Representative images of mammosphere were taken (top-right) (scale bar 400 μm), and mammosphere larger than 50µm were counted and mammosphere formation efficiency was quantified. Quantified graph showing primary mammosphere formation efficiency of HCI-10 cells stably expressing control or KLF8 shRNA. Primary mammosphere were collected and cultured again in mammosphere culture condition for 5- days to form secondary mammosphere (bottom). (Student t test reported as mean ± SEM, *p<0.05, and **p<0.01. [file Image_1.jpeg]

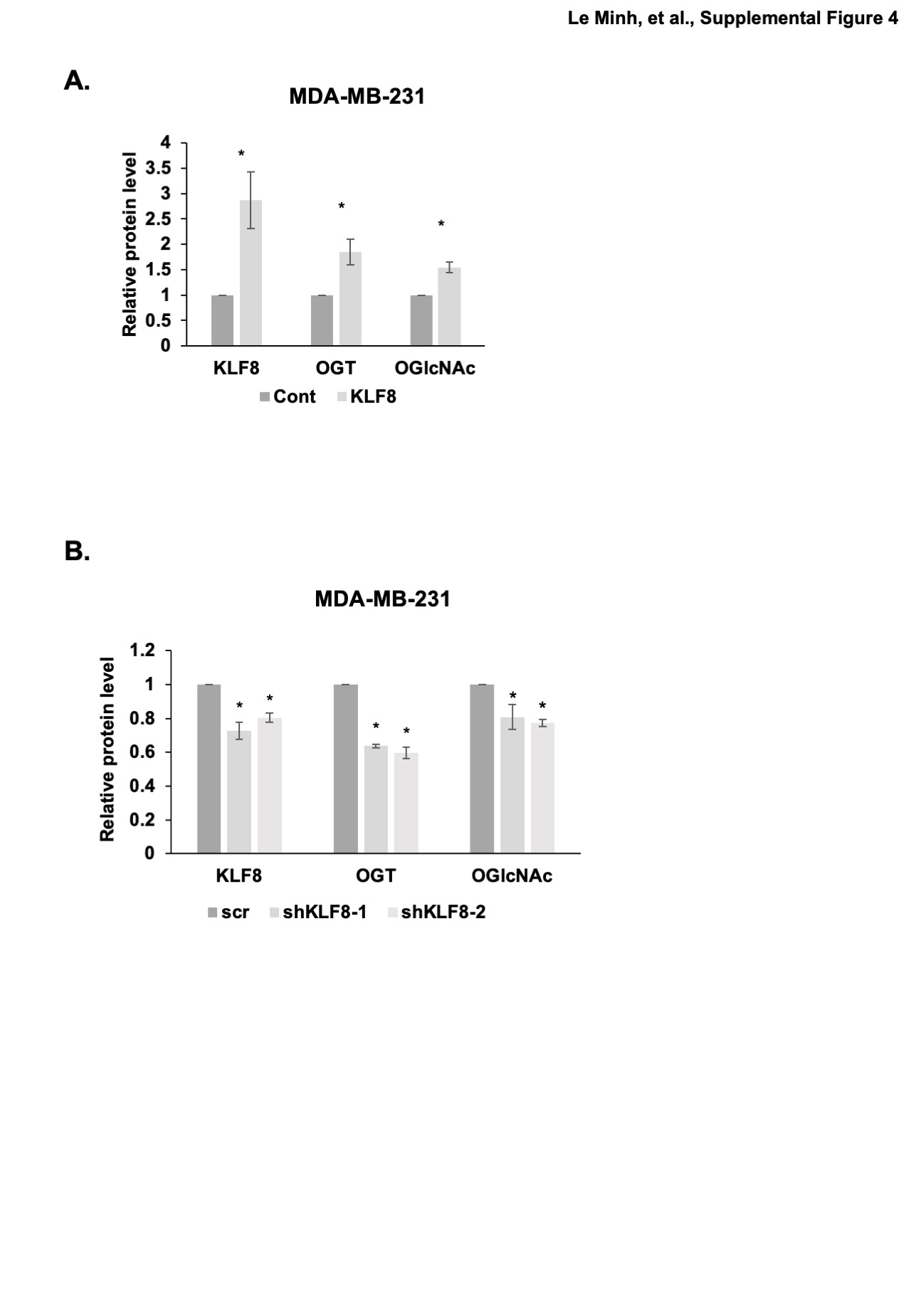

Supplement: Supplementary Figure 2 — KLF8 regulates expression of stem cell markers in breast cancer cells. (A) Lysates from SUM159 cells stably overexpressing control or KLF8 were collected for immunoblot analysis of CSCs markers using indicated antibodies (left), and quantified graph of relative level of CSCs detected by immunoblot from SUM159 cells control or with KLF8 overexpression (right). (B) Quantified graph showing relative mRNA level of CSCs markers as detected by qRT-PCR using probes against genes in SUM159 cells stably overexpressing control or KLF8. (C) Lysates from SUM159 cells stably expressing control or KLF8 shRNA were collected for immunoblot analysis of CSCs markers using indicated antibodies (left), and quantified graph of relative level of CSCs levels (right). (D) Quantified graph showing relative mRNA level of CSCs markers as detected by qRT-PCR using probes against indicated targets in SUM159 cells stably expressing control or KLF8 shRNA. Student t test reported as mean ± SEM, *p<0.05. [file Image_2.jpeg]

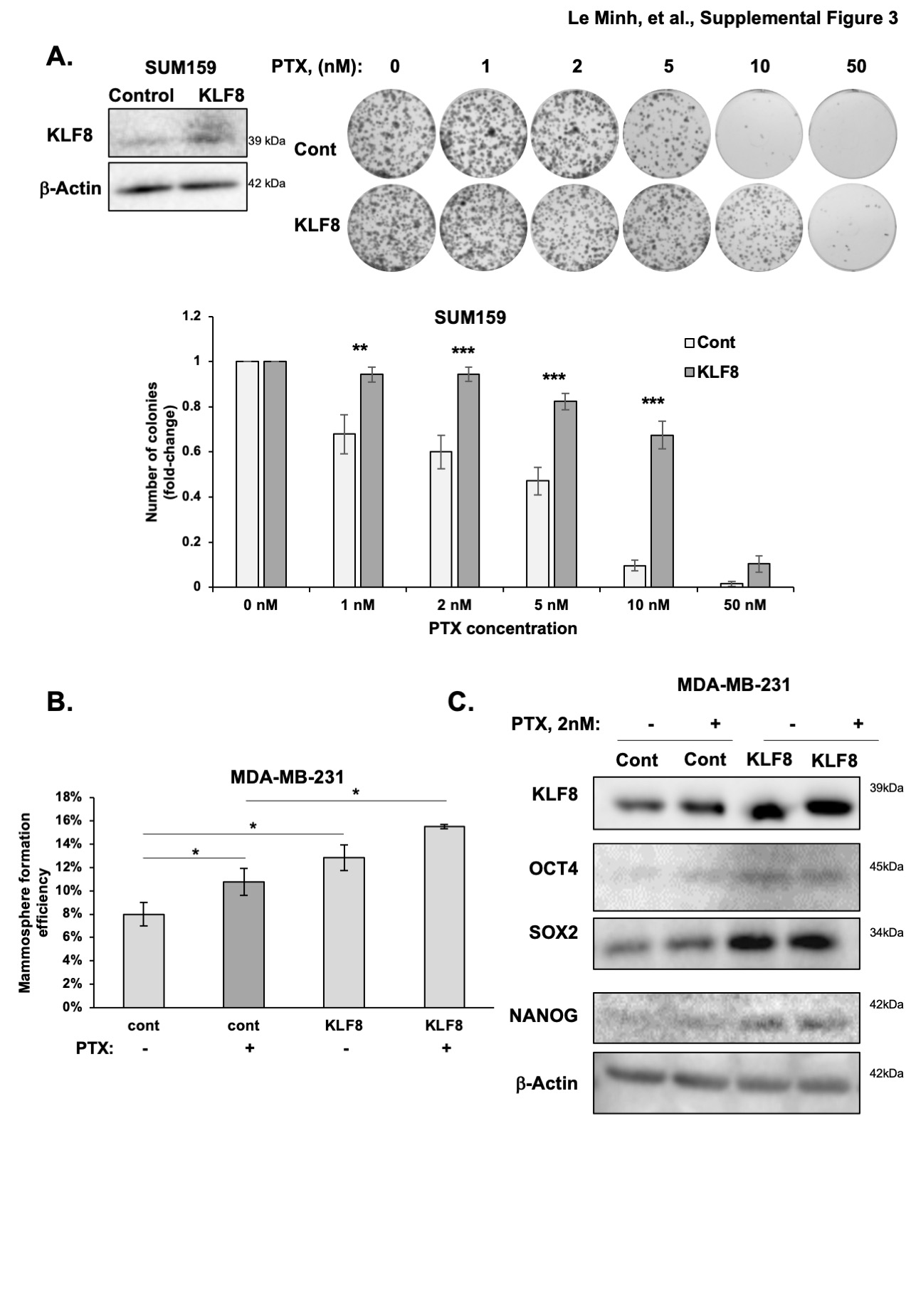

Supplement: Supplementary Figure 3 — KLF8 promotes resistance to paclitaxel in breast cancer cells in vitro. (A) Lysates of SUM159 cells overexpressing control or KLF8 were collected for immunoblot using indicated antibodies (top-left). SUM159 cells overexpressing control or KLF8, treated with increasing dose of paclitaxel for 48h, were grown in clonogenic assay for 10-14 days. Colonies were stained, counted. Representative images show stained colonies formed in the clonogenic assay after 10-14 days from SUM159 cells control or with KLF8 overexpression, treated with increasing dose of paclitaxel for 48h (top-right). Quantified graph of counted colonies after 10-14 days from SUM159 cells control or with KLF8 overexpression, treated with increasing dose of paclitaxel for 48h, in clonogenic assay (bottom). Two-way ANOVA with Holm-Sidak test reported as mean ± SEM, **p<0.01. ***p<0.001. (B) MDA-MB-231 cells control or stably overexpressing KLF8 were treated with 2nM final concentration of Paclitaxel for 48h and cells were cultured in mammosphere formation assay for 5-7 days. Mammosphere larger than 50 µm were counted. (C) MDA-MB-231 cells control or stably overexpressing KLF8 were treated with 2nM final concentration of Paclitaxel for 48h and cell lysates were collected for immunoblot analysis using indicated antibodies. [file Image_3.jpeg]

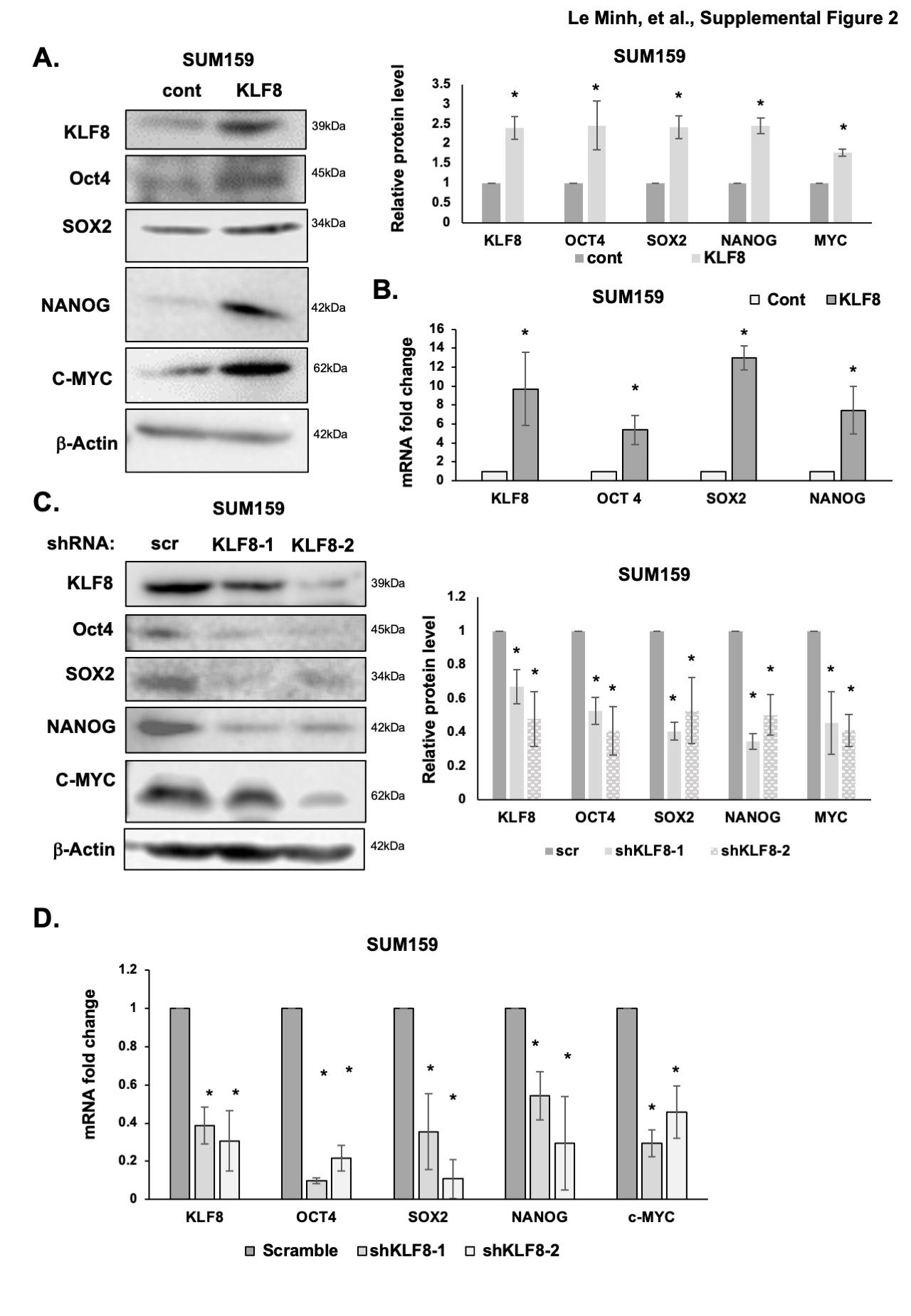

Supplement: Supplementary Figure 4 — (A) Quantified graph showing relative protein level of KLF8, OGT, and O-GlcNAc in MDA-MB-231 cells control or overexpressing KLF8 as detected by immunoblot using indicated antibodies (corresponding to ). Student t test reported as mean ± SEM, *p<0.05. (B) Quantified graph showing relative protein level of KLF8, OGT, and O-GlcNAc in MDA-MB-231 cells expressing control shRNA or KLF8 shRNA as detected by immunoblot using indicated antibodies (corresponding to ). Student t test reported as mean ± SEM, *p<0.05. [file Image_4.jpeg]

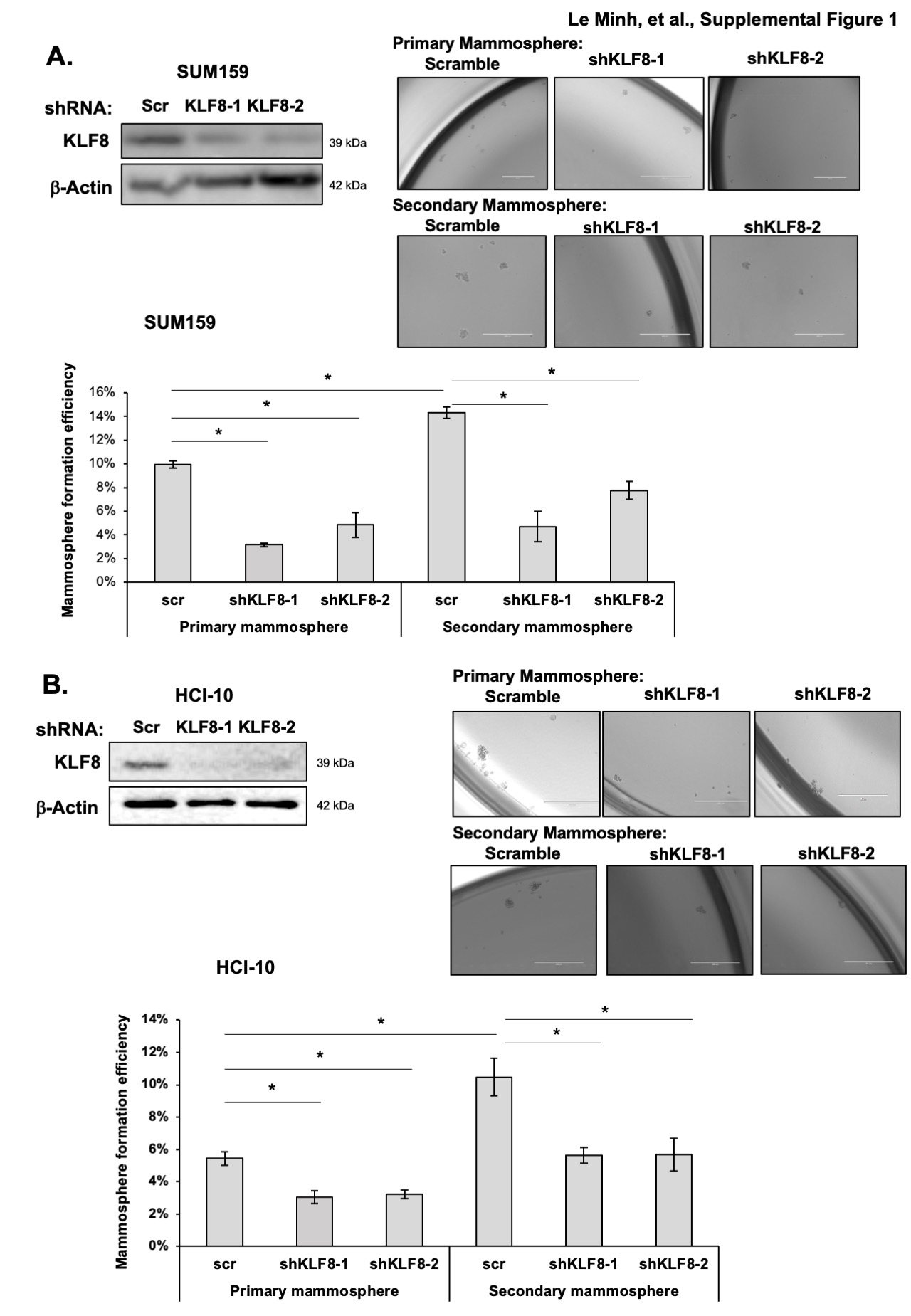

Supplement: Supplementary Figure 5 — Increased KLF8 expression associates with poor outcome of breast cancer. (A) Kaplan-Meier plot showing overall survival of all breast cancer patients with different level of KLF8 mRNA. (B) Kaplan-Meier plot showing overall survival of basal-type breast cancer patients with different level of KLF8 mRNA. [file Image_5.jpeg]
